# Serum Cholesteryl Ester Transfer Protein (CETP) and Sortilin (SORT) in Patients with Psoriasis with Relation to Systemic Treatment

**DOI:** 10.3390/metabo12040340

**Published:** 2022-04-09

**Authors:** Julia Nowowiejska, Anna Baran, Julita A. Krahel, Tomasz W. Kamiński, Magdalena Maciaszek, Iwona Flisiak

**Affiliations:** 1 Department of Dermatology and Venereology, Medical University of Bialystok, Zurawia 14 St., 15-540 Bialystok, Poland; anna.baran@umb.edu.pl (A.B.); julita.leonczuk@gmail.com (J.A.K.); iwona.flisiak@umb.edu.pl (I.F.); 2 Pittsburgh Heart, Lung and Blood Vascular Medicine Institute, University of Pittsburgh, Pittsburgh, PA 15260, USA; kamins1@pitt.edu; 3 Department of Infectious Diseases and Hepatology, Medical University of Bialystok, Zurawia 14 St., 15-540 Bialystok, Poland; mm.maciaszek@wp.pl

**Keywords:** cholesteryl ester transfer protein (CETP), sortilin (SORT), psoriasis, metabolic syndrome, dyslipidemia, inflammation, methotrexate, acitretin

## Abstract

Psoriasis is a common inflammatory skin disease, which is tightly associated with metabolic disorders. Cholesteryl ester transfer protein (CETP) and sortilin (SORT) are molecules engaged in lipid metabolism of proatherogenic properties. They have been hardly ever studied in psoriasis before. Serum CETP and SORT concentrations were measured in 33 patients with plaque-type psoriasis before and after 12 weeks of treatment with methotrexate or acitretin. There was no significant difference in CEPT and SORT serum concentrations between patients and controls. Positive correlations between CETP after the treatment with acitretin and activity of transaminases (R = 0.65, R = 0.56, respectively) were noted. CETP was positively related with triglycerides (R = 0.49), glucose (R = 0.54) and CRP (R = 0.64) before the treatment with methotrexate, which all disappeared afterwards. Systemic therapy with methotrexate caused normalization of SORT concentration. There was significant correlation between SORT and WBC (*p* < 0.01) and CRP (*p* < 0.05). CETP and SORT cannot be used as individual biomarkers. Nevertheless, they show some interesting relations with other parameters. Increased concentration of CETP perhaps could investigated as a marker of liver side effects of acitretin treatment in psoriatics. SORT could be considered as a new indicator of metabolically induced inflammation in psoriasis. Methotrexate may be preferred in patients with high SORT concentrations. Further studies are needed to establish their exact role in psoriatic patients.

## 1. Introduction

Psoriasis is one of the most common chronic skin diseases. It is estimated that it affects 2–4% of the worldwide population [1]. The etiopathogenesis of psoriasis has not been fully understood. It is suspected that genetic factors, as well as autoimmune mechanism are involved, which are additionally modified by environmental factors [2]. The essence of psoriasis is the hyperproliferation of epidermal cells and parakeratosis, which leads to erythematous-infiltrative, scaly plaques [3]. Nowadays, psoriasis should be acknowledged as a kind of systemic disease associated or leading to development of many other disorders and shortening the patients’ life expectancy to about 5 years [4]. Some of the most important comorbidities of psoriasis are undoubtedly metabolic disorders [5]. Many investigations aimed to study this relationship, some of them with conflicting results, nevertheless majority of research has proved psoriasis to be related with obesity, arterial hypertension, dyslipidemia or diabetes mellitus (DM), and metabolic syndrome (MS) [5,6]. To highlight the association between chronic inflammatory condition in psoriasis with metabolic disturbances, even a term called ‘metaflammation’ has been introduced [7]. Psoriatics are known to have lipid profile aberrations and increased risk of cardiovascular incidences that could result in death [4,5]. What is interesting, associations between the severity of psoriatic skin lesions and intensity of these metabolic disorders have been widely proved [7] and the risk of cardiovascular disease seems to be elevated especially in patients with severe psoriatic skin lesions [6]. Moreover, some drugs used in the treatment of dyslipidemia or carbohydrate metabolism disorders were also reported to have beneficial impact on psoriatics’ skin condition [5,7].

Cholesteryl ester transfer protein (CETP) is a glycoprotein which takes part in intravascular high-density lipoprotein (HDL) metabolism. CETP is involved in transfer of cholesteryl esters chains and triglycerides (TG) between the HDL fraction and the non-HDL fraction (very low-density lipoproteins, VLDL, and low-density lipoproteins, LDL) [8,9]. Due to such properties, CETP is considered a proatherogenic protein since it leads to elevation of LDL and decrease of HDL at the same time [8,9]. Obviously CETP concentration is increased in subjects with dyslipidemia [10]. *CETP* gene in humans is located on chromosome 16 and studies on subjects with *CETP* genes polymorphism have showed that persons whose genotypes resulted in CETP inhibition had lower risk of coronary artery disease [11].

Sortilin (SORT) is a transmembrane, multi-ligand receptor, which is considered among the Vacuolar protein sorting 10 protein (Vps10p) domain receptor family [9]. SORT probably participates in glucose metabolism and may have influence on DM development [9,12]. SORT was also proved to be engaged in regulatory processes of cell proliferation [13], and due to its associations with p75 neutrophin receptor (P75NTR), is involved in apoptotic processes [14].

CETP and SORT have been hardly ever studied in psoriatic patients before, so the available data is scarce. Considering psoriasis is a serious medical, social and economic problem [15], it should be investigated in relevance to compounds that could potentially become markers of psoriasis comorbidities, complications and indicate the choice of therapy. This is why we analyzed the influence of antipsoriatic therapy on investigated markers and their correlations with metaflammation indicators, which could potentially point into their specific use in daily clinical practice. SORT and CETP may perhaps serve as such markers considering their role in metabolic disorders, which are undoubtedly associated with psoriasis.

## 2. Results

The baseline parameters of enrolled patients and controls are presented in Table 1. There was no significant difference between patients and controls in terms of sex and age (NS). After administered treatment psoriasis severity assessed in PASI significantly decreased (patients’ condition improved) (Table 1b).

### 2.1. CETP

**Figure 1:**
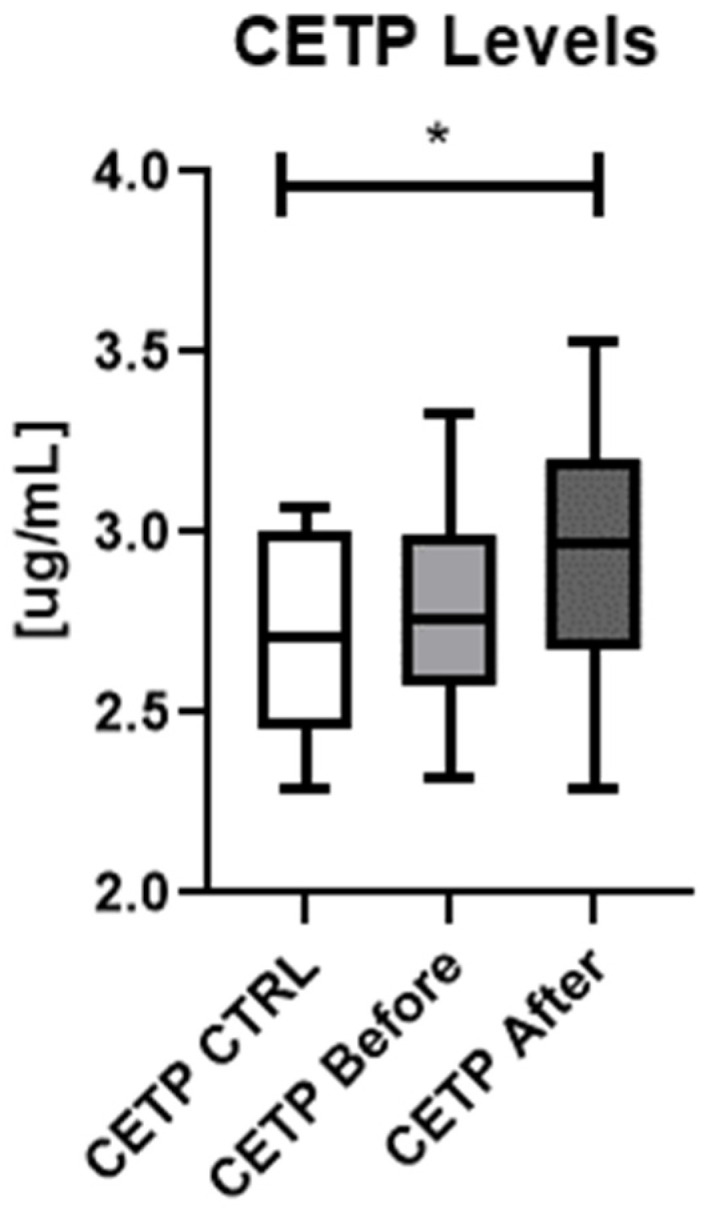
Concentrations of CETP in patients (before and after the treatment) and controls. * means *p* < 0.05 when comparing all three groups using ANOVA-test.

There was no significant difference in CETP serum concentrations between the patients and controls (NS) and in patients before and after the treatment (NS) but there was a statistically significant difference in CETP concentration between all three analyzed groups achieved in ANOVA (*p* < 0.05) (Figure 1).

**Figure 2:**
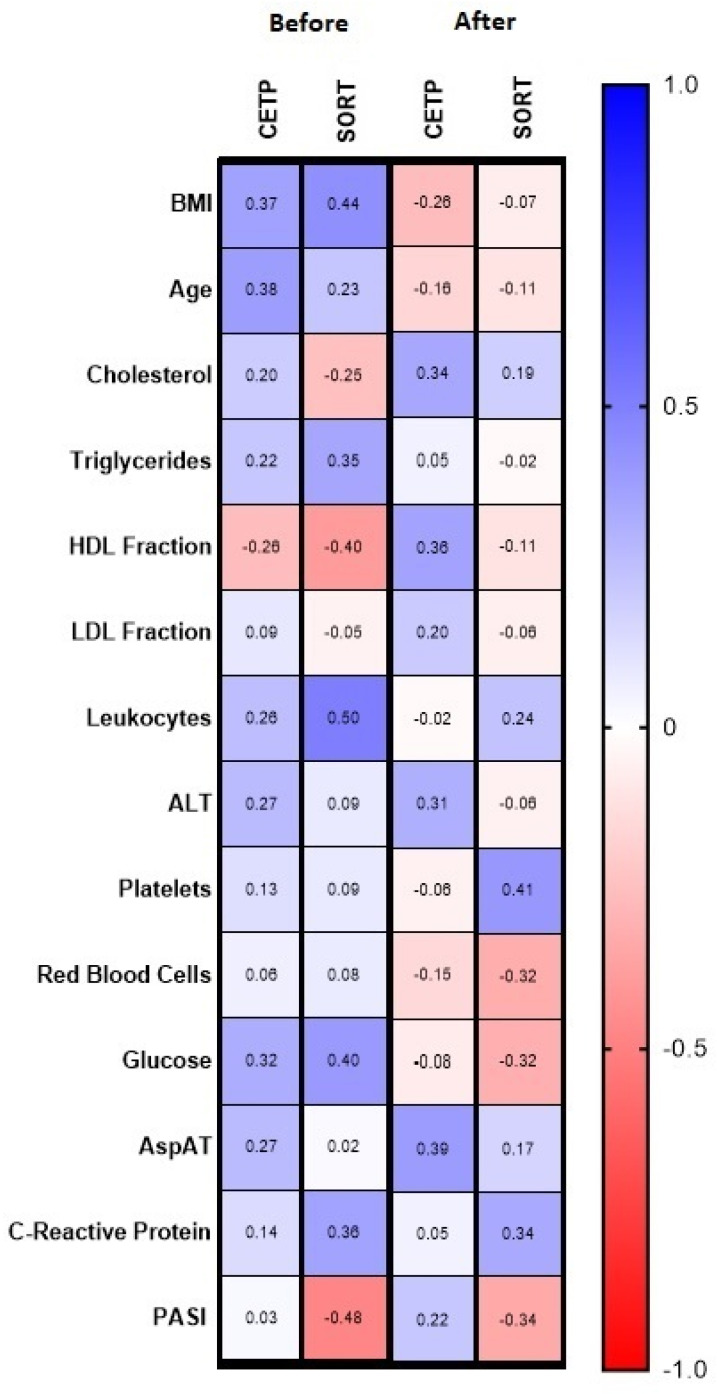
Associations between CETP, SORT, PASI and laboratory parameters before and after the treatment.

Positive correlations between CETP and BMI, age and glucose level were noted before the treatment (R = 0.37, R = 0.38, R = 0.32, respectively, Figure 2). Moreover, after the treatment there was a weak correlation between CETP and total cholesterol concentration (R = 0.34) and HDL concentration (R = 0.36), and also between CETP and AST activity (R = 0.39), along with a trend for ALT concentration (R = 0.31).

There was no correlation between the CETP concentration and PASI (NS) both before and after therapy.

**Figure 3:**
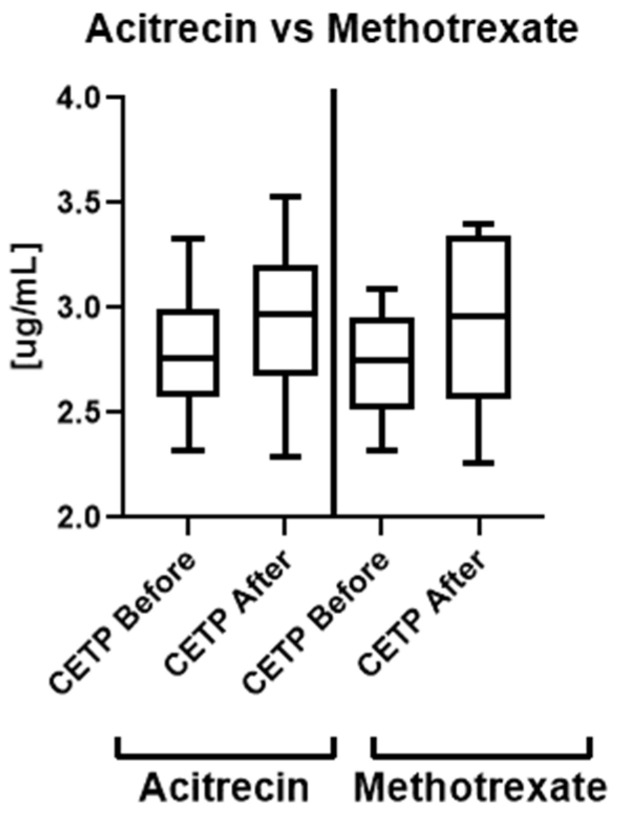
Concentrations of CETP in patients before and after the treatment with acitretin and methotrexate.

There was no significant difference in serum CETP level before and after the treatment (NS) (Figure 1), including depending on the chosen systemic antipsoriatic agent (methotrexate or acitretin) (NS) (Figure 3).

**Figure 4:**
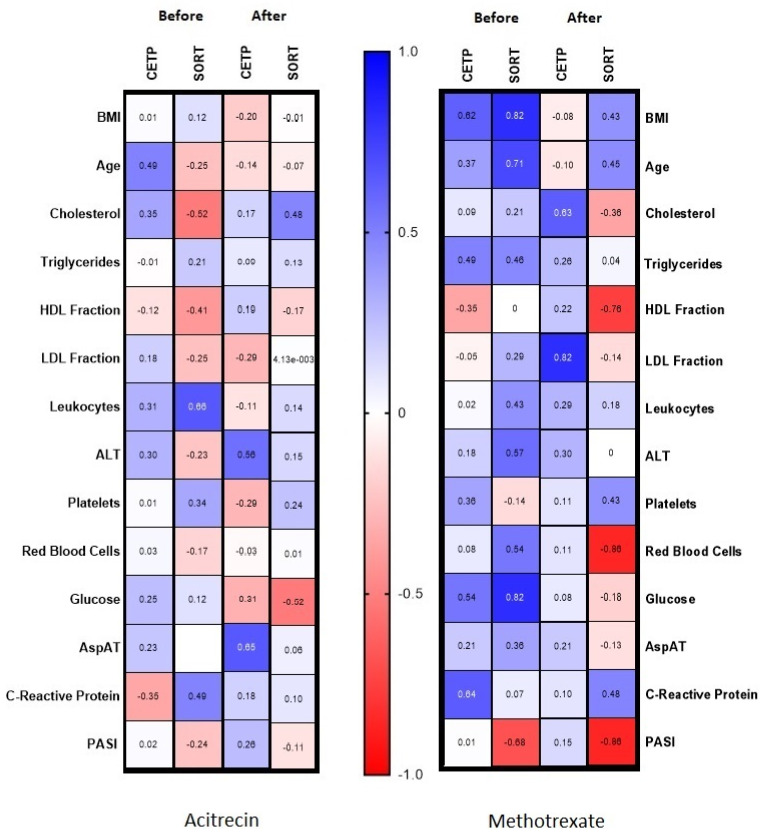
Correlations of SORT and CETP in acitretin and methotrexate groups with the basal parameters before and after treatment.

After the treatment, in general, we observed a positive correlation between CETP and AST (R = 0.39) and a trend for ALT (R = 0.31) (Figure 2). There was a positive correlation between the CETP concentration after the treatment with acitretin and the activity of AST and ALT (R = 0.65; R = 0.56, respectively, Figure 4). In the group of patients treated with methotrexate we found a positive correlation between CETP concentration before the treatment and triglycerides (R = 0.49), glucose (R = 0.54) and CRP (R = 0.64), which all disappeared afterwards (Figure 4). In the same group, after the treatment, a positive correlation with total cholesterol concentration (R = 0.63) and LDL (R = 0.82 were noted, Figure 4).

### 2.2. Sortilin

**Figure 5:**
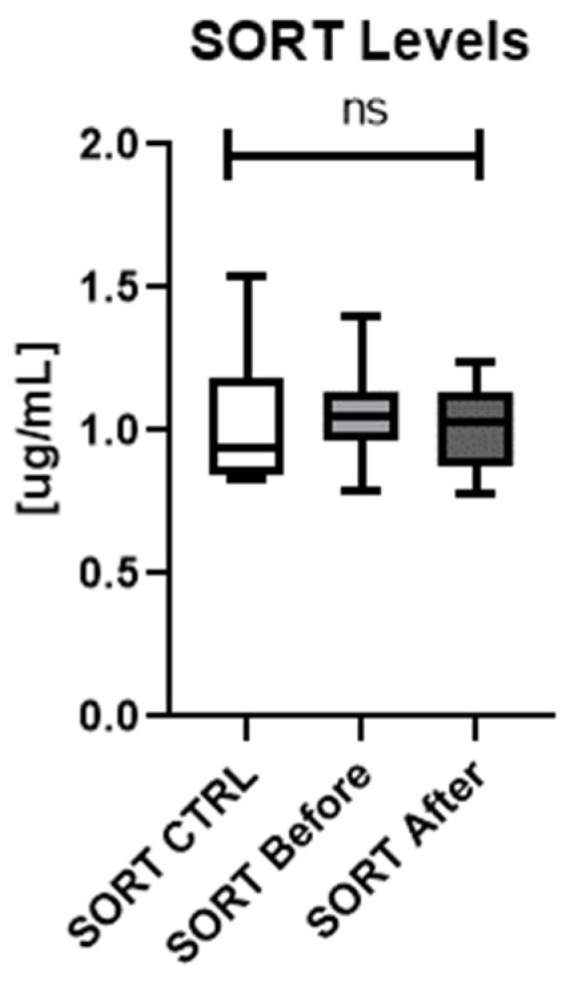
Concentrations of SORT in patients (before and after the treatment) and controls.

Serum SORT concentration was higher in patients than in controls, although the difference was not significant (ns). There was no significant difference in SORT concentrations in patients before and after the treatment (ns) (Figure 5).

There was a negative correlation between SORT and PASI (R = −0.48) and a positive between SORT and BMI (R = 0.44) (Figure 2).

Among the biochemical parameters there was a significant association between SORT concentration and inflammation indicators—positive correlation with WBC (R = 0.5) and trend for CRP (R = 0.36) before the treatment (Figure 2). Moreover, these correlations disappeared during the treatment with acitretin (Figure 4). We also noted a positive correlation of SORT with glucose (R = 0.4) and trend for triglycerides (R = 0.35) and a negative correlation with HDL concentration (R = −0.4) before the treatment (Figure 2).

**Figure 6:**
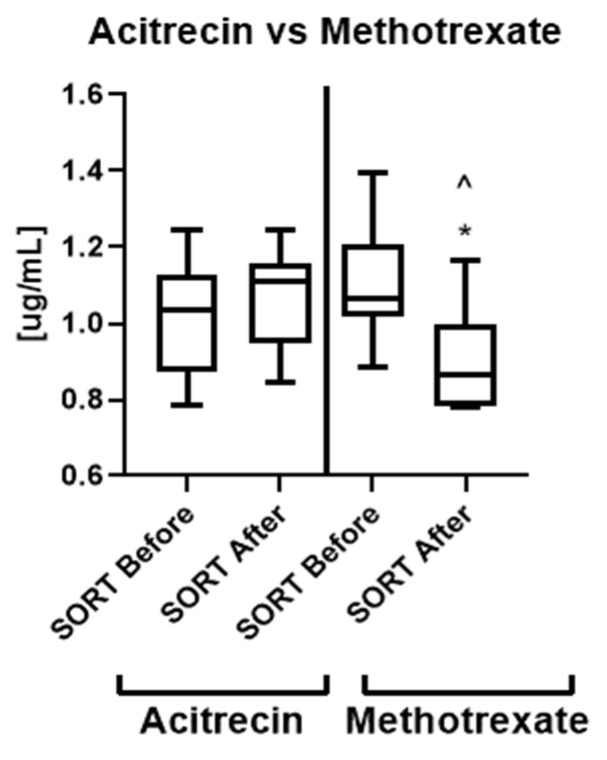
Concentrations of SORT in patients before and after the treatment with acitretin and methotrexate. * means statistically significant difference in SORT concentration on acitretin vs. methotrexate after treatment; ^ means statistically significant difference in SORT concentration before and after treatment in methotrexate subgroup.

The analysis of the influence of particular antipsoriatic systemic drug revealed that methotrexate caused significant decrease in SORT concentration, which became similar to the one observed in controls (Figure 6). Methotrexate caused statistically significant drop in SORT concentration, which was significantly lower than before the treatment and also comparing to the levels after acitretin treatment (Figure 6).

## 3. Discussion

Psoriasis is nowadays perceived as a systemic inflammatory condition characterized by many comorbidities. Although plenty of markers of metabolic disorders have been proved to be also significant in psoriasis, further intensive searching for newer ones is still performed. There is no doubt that psoriatics suffer from aberrations in lipid metabolism but the particular alterations in lipid components sometimes differ between the studies, which makes it difficult to interpret in context of pointing a universal biomarker [16]. Considering the increased risk of cardiovascular complications and death in psoriatics, as well as complications associated with long-time antipsoriatic therapy, we decided to explore the role of CETP and SORT in psoriatic patients with hope for interesting findings that could be further investigated for potential use in daily clinical practice. Noteworthy, the data on these molecules in psoriatics are very limited, hence it is not an easy task to analyze our results and draw adequate conclusions. Nevertheless, we are one of the first to report information of possible role of CETP and SORT in such patients, which might initiate further research. As far as we are concerned, they have never been studied in other skin diseases, except for one newest paper by Waśkiel-Burnat et al. where SORT has been investigated in subjects with alopecia areata [17]. 

We managed to find one study which evaluated serum CETP concentration in psoriatics. Torkhovskaia et al. interestingly found decreased serum CETP level and concluded that there are alterations in reverse cholesterol transport in psoriasis, which may affect cell supply with cholesterol and cells proliferation in consequence [18]. Indeed, research show that inflammatory condition, including psoriasis, may lead to changes in lipoprotein metabolism and reverse cholesterol transport pathway [16]. Therefore, although CETP is a known marker of dyslipidemia and its elevated concentrations are observed in such individuals, and although psoriatics are also in increased risk of this disorder, CETP status in such patients may not be easy to establish. We surprisingly did not observe significantly higher CETP concentration in patients comparing to controls. Perhaps other confounders or modifiers take part in that interplay. The role of CETP is still not completely clear because it is suspected that it may also have other nonlipid transfer properties [11], so its true role in psoriasis is yet to be discovered and may be much more complex. We also need to keep in mind the limitation of our study which is small number of subjects involved.

We did not observe any correlation between the psoriasis severity and CETP concentration, but we managed to find a positive correlation with age. Before the administered treatment we did not notice any significant correlations of CETP with laboratory parameters. However, within the subgroups on certain drugs some relations were noted.

The analysis of the influence of treatment on CETP concentration did not reveal any beneficial outcome, including that particular antipsoriatic systemic drug did not show advantage in administration of one compared to the other. Nevertheless, we also noticed positive correlation between CETP concentration before the treatment with methotrexate and glucose, triglycerides and CRP concentration, which disappeared afterwards. This may indicate that this drug could be preferred in psoriatics with intense inflammatory condition, carbohydrates metabolism disorders and hypertriglyceridemia especially in order to modify or lower the further metabolic complications. Furthermore, CETP seems to exert a protective role in these disorders development in psoriasis. We found positive correlation between the liver function enzymes activity and CETP concentration after the treatment with acitretin, which is known to negatively affect this organ. Therefore, a potential idea for future research is to consider CETP as a marker of the side effects of acitretin in psoriatics.

As for SORT investigation, its serum concentration has not been studied in psoriatics before. The only study was the assessment of SORT expression in psoriatic epidermis, which was significantly increased [14]. In our study SORT serum concentration did not differ significantly between patients and controls and did not change after the treatment, although it perhaps should be verified on larger cohorts.

The second area of SORT involvement is apoptosis. In our previously published review paper [9], we made a suggestion that SORT may actually be an apoptosis-promoting agent in psoriasis. As we found a negative correlation between the SORT concentration and PASI, we may assume that SORT could become a part of compensatory mechanism in psoriasis. Therefore, in patients with low SORT concentration we observed high PASI (severe skin lesions) and in patients with high SORT concentration–low PASI (less severe skin lesions).

As for laboratory parameters, SORT positively correlated with glucose and triglycerides concentration and negatively with HDL. In addition to the positive correlation of SORT and BMI, we may suspect that SORT could be studied as a marker of metabolic complications in psoriatics but still not used as a solitary indicator.

Another interesting finding is that SORT is positively correlated with inflammation indicators, which perhaps points the protein as a novel potential indicator of metabolically induced inflammatory condition in psoriatics. As these dependencies disappeared after treatment with acitretin, we presume it could be the preferred drug in subjects with high inflammatory markers, which should be proceeded in further studies.

An important observation from our study is that methotrexate caused statistically significant decrease in SORT concentration, which was significantly lower than before the treatment, therefore we believe that this antipsoriatic agent could be considered as the drug of choice in patients with elevated SORT concentration. Our results are preliminary, but furthermore significant links between the protein and metabolic disorders indicators noted additionally prove the broad protective cardiometabolic effects of methotrexate.

As for limitations of our study, it was a single-center research with relatively small number of subjects enrolled. Furthermore, all of the presented results should be interpreted with a higher than usual level of criticism since the sample size and the statistical power of the presented study is relatively low. We are aware that our outcomes should be treated as preliminary but they may point out a potential direction for future studies.

In the future we would like to extend our study and introduce some dietary changes and physical activity to the participants and observe their influence on the investigated proteins. Moreover, it would be valuable to study the impact of hypolipidemic and hypoglycemic drugs on analyzed proteins.

## 4. Materials and Methods

The study enrolled 33 patients (12 females and 21 males) with a flare of plaque-type psoriasis, at median age of 51 (19–78) years old and compared them with 18 sex-matched and age-matched volunteers without skin diseases. All participants signed informed written consents before initiation. None of the patients or controls was under any dietary restriction or was taking medications for at least three months before the enrollment. The exclusion criteria comprised of other types of psoriasis, chronic inflammatory diseases and cardiometabolic, autoimmune or oncological comorbidities. Psoriasis area and severity index (PASI) was assessed by the same person in all patients. The investigated group was divided depending on the disease intensity into three sub-groups: mild (PASI I) < 10 points, moderate (PASI II) between 10 and 20, and severe (PASI III) > 20. Body mass index (BMI) was calculated as weight/height^2^ (kg/m^2^). Laboratory tests including C-reactive protein (CRP), serum glucose, total cholesterol (Total Chol), HDL (high-density lipoprotein), LDL (low-density lipoprotein), triglycerides (TGs), and transaminases: alanine and asparagine (AST, ALT) were performed before the treatment. The patients received two systemic treatment options: 14 persons received methotrexate (15 mg/week) using folic acid supplementation (15 mg/week, 24 h after methotrexate intake) and 19 subjects were started on acitretin at a dose 0.5 mg/kg/day. The treatment period lasted 12 weeks. The study was approved by the Bioethical Committee of Medical University in Bialystok (no R-I-002/429/2017) and in accordance with the principle of the Helsinki Declaration. 

### 4.1. Serum Collection

Fasting blood samples were received from subjects without dermatoses who served as control group and from patients before and after 12 weeks of treatment with antipsoriatic agent using vacutainer tubes with a clot activator. Samples were centrifuged at 2000× *g* for 10 min and preserved at −80 °C until analyses. CETP and SORT concentrations were measured using an enzyme immunoassay kit supplied by Cloud Clone, SEC895Hu and SEA814Hu. Optical density was read at a wavelength of 450 nm. The concentrations were measured by interpolation from calibration curves prepared with standard samples supplied by the manufacturer. All the tests were performed by the same person in standardized laboratory settings. 

### 4.2. Statistical Analysis

The normally distributed data (Gaussian conditions met) were presented as mean ± SEM, while the non-Gaussian data was presented as median 25th–75th percentile and full-range. Normality of distribution was tested using a Shapiro–Wilk W test. The Student *t*-test or nonparametric Mann–Whitney test were used to compare differences between the psoriasis group and the control group. The chi-square test was used for categorical variables. The correlations were analyzed using Spearman’s Rank correlation analysis. A two-tailed *p* < 0.05 was considered statistically significant. Computations were performed using GraphPad 8 Prism (GraphPad Software; La Jolla, CA, USA). 

## 5. Conclusions

We are one of the first to report on possible role of CETP and SORT serum concentration in the management of psoriasis. As both CETP and SORT were not staistically different between patients and controls, it seems that they cannot be used as individual biomarkers. Nevertheless, they show some intersting relations with other parameters that could further investigated on larger cohorts. Increased concentration of CETP could be investigated as a marker of liver side effects of acitretin in psoriatics. SORT could be studied as a new indicator of metabolically induced inflammatory condition in psoriasis and perhaps acitretin could be considered as a drug of choice in patients with elevated inflammatory markers. Methotrexate may be preferred in patients with high SORT concentrations. Our research may point into some role of CETP and SORT in antipsoriatic treatment administration, which surely requires further investigation.

## Figures and Tables

**Table 1:** Baseline characteristics of patients and controls.

**(a)**		
**Parameter**	**Controls (n = 16)**	**Patients (n = 33)**
Sex (M/F)	8/8	21/12 NS
Age [years]	38.56 ± 3.69	46.6 ± 3.25 NS
Height [cm]	172.4 ± 2.45	174.6 ± 1.56
Weight [kg]	**73.1 ± 3.79**	83.8 ± 2.88 *
BMI	**24.36 ± 0.93**	27.5 ± 0.95 *
NS, non-significant; M/F, male/female ratio; BMI, body mass index		
**(b)**		
**Parameter**	**Values before**	**Values after**
PASI	**16.27 ± 1.06**	10.32 ± 0.64 ***
RBC (×10^3^/mL)	4.61 ± 0.11	4.68 ± 0.11
PLT (×10^3^/mL)	228.9 ± 11.17	232.5 ± 9.82
WBC (×10^3^/mL)	7.38 ± 0.35	6.93 ± 0.31
Glucose (mg/dL)	91.61 ± 5.11	92.36 ± 5.31
Total cholesterol (mg/dL)	167.3 ± 4.91	178.5 ± 5.56
Triglycerides (mmol/L)	121.6 ± 8.83	*151.4 ± 12.63* (*p* = *0.061*)
HDL (mmol/Ll)	46.92 ± 2.13	47.12 ± 3.41
LDL (mmol/L)	100.5 ± 3.99	103.5 ± 4.3
CRP (mg/L)	**7.33 ± 2.01**	3.21 ± 0.68 *
ALT (U/L)	22.45 ± 2.55	22.79 ± 3.75
AST (U/L)	24.48 ± 2.41	23.97 ± 3.48

*/*** and **bold font** means statistically significant difference between controls and patients with *p* < 0.05/0.001 respectively; *italic* font means the existence of a trend; PASI, psoriasis area and severity index; RBC, red blood cells; PLT, platelets; WBC, white blood cells; TGs, triglycerides; HDL, high-density lipoproteins; LDL, low-density lipoproteins; CRP, C-reactive protein; ALT, alanine transaminase; ASPAT, asparagine transaminase.

## Data Availability

Because of the participant consent obtained as part of the recruitment process, it is not possible to make these data publicly available. The data resented in this study are available on request from the corresponding author.
